# Supplementary material for: Comparative analysis of gene importance in Escherichia coli across growth conditions
Source: mSystems. 2026 Apr 14;11(5):e01425-25. doi: 10.1128/msystems.01425-25 (PMC13185578; doi:10.1128/msystems.01425-25)
Supplement: Supplemental material — Supplemental figures and captions for supplemental tables. [file msystems.01425-25-s0001.pdf]

Supplementary material for:

Comparative Analysis of Gene Importance in Escherichia coli Across Growth Conditions

Antoine Champie[1*], Simon Jeanneau[1*], Amélie De Grandmaison[1*], Mathias Martin Silva[1], Jean-Philippe Coté[1], Pierre-Étienne Jacques[1,2,3,†], Sébastien Rodrigue[1,2,3,†]

Supplementary Figures:

- Supplementary Figure 1: Number of genomic **insertions per day in each tested medium.**
- Supplementary Figure 2: **Visual representation of the *fi*Score** analysis procedure.
- Supplementary Figure 3: **Repartition of genes according to their *fi*Score** across all passages and growth media.
- Supplementary Figure 4**: Determination of "essential" genes using exponential fitting of the** non-**essential** gene p**opulation**.
- Supplementary Figure 5**: Validation of the *fi*Score** method and comparison with Bio-Tradis.
- Supplementary Figure 6: **Multidimensional classification of gene fi**tness impact across passages based on read **abundance and depletion dynamics.**
- Supplementary Figure 7: **Competition assay between** mutants with different status and BW25113 wild type.
- Supplementary Figure 8: Importance of the genes in the amino acid metabolism pathway in defined media.
- Supplementary Figure 9. **Number and localization of the transposon insertions in isolated mutants.**
- Supplementary Figure 10. Probability of observing **≤ 2 insertions** in bins of varying length.

Supplementary Tables:

- Supplementary_**Table_1_TnSeq_Statistics**
  - Number of read and mapped **insertion for each time point.**
- Supplementary_Table_2_Genes_***fi***Score
  - ***fi***Score of all genes in all 3 media.
- Supplementary_**Table_3_Model_Predictions**
  - Fitness **prediction** made using the iML1515 metabolic model.
- Supplementary_Table_4_**Enriched_functions**
  - **Enriched KEGG functions in** each primary important group of gene and in the modules.
- Supplementary_**Table_5_Partial_Genes**
  - **Description of g**enes harboring a **fi**tness-**impacting sector**.
- Supplementary_Table_6_Data_Supp_slopes
  - Gene summary with read density, fold-change, and regression metrics.
- Supplementary_Table_7_Modules
  - List of all genes composing both modules.

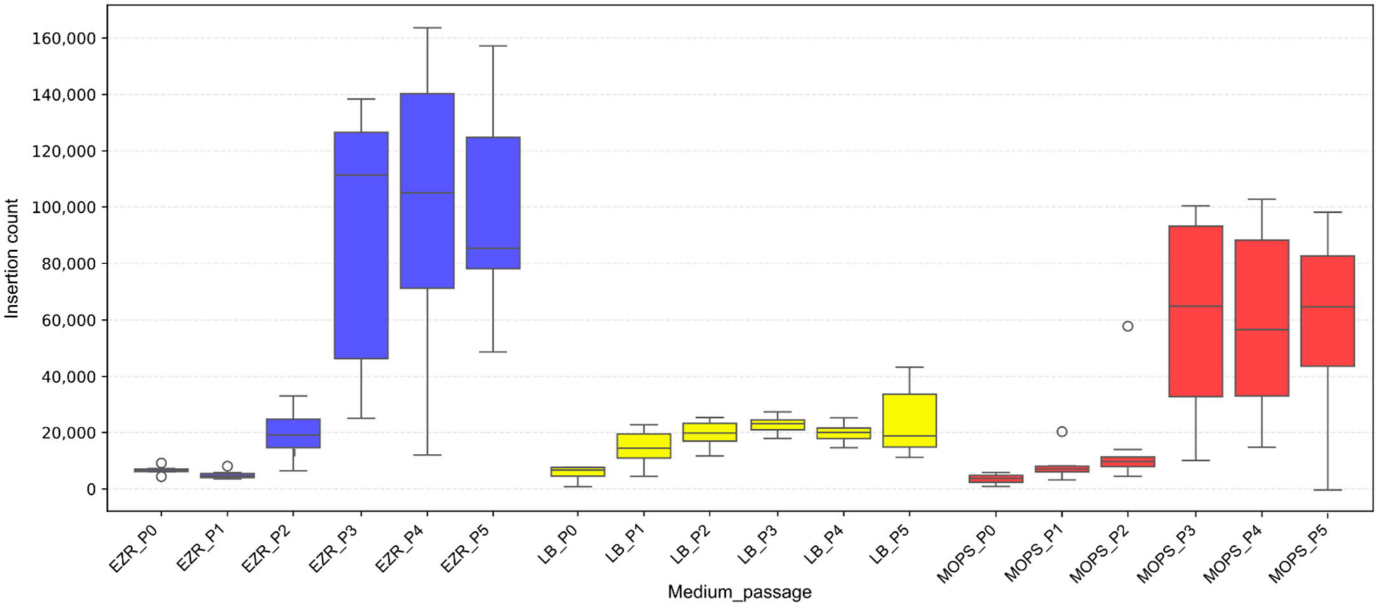

Supplementary Figure 1: Number of genomic insertions per passage in each tested medium. The Y-axis represents the number of insertions for all replicates on each passage before merging for analysis. Boxes represent the 25th–75th percentiles with the median indicated by a horizontal line. Whiskers show the minimum and maximum values. Individual dots indicate outliers

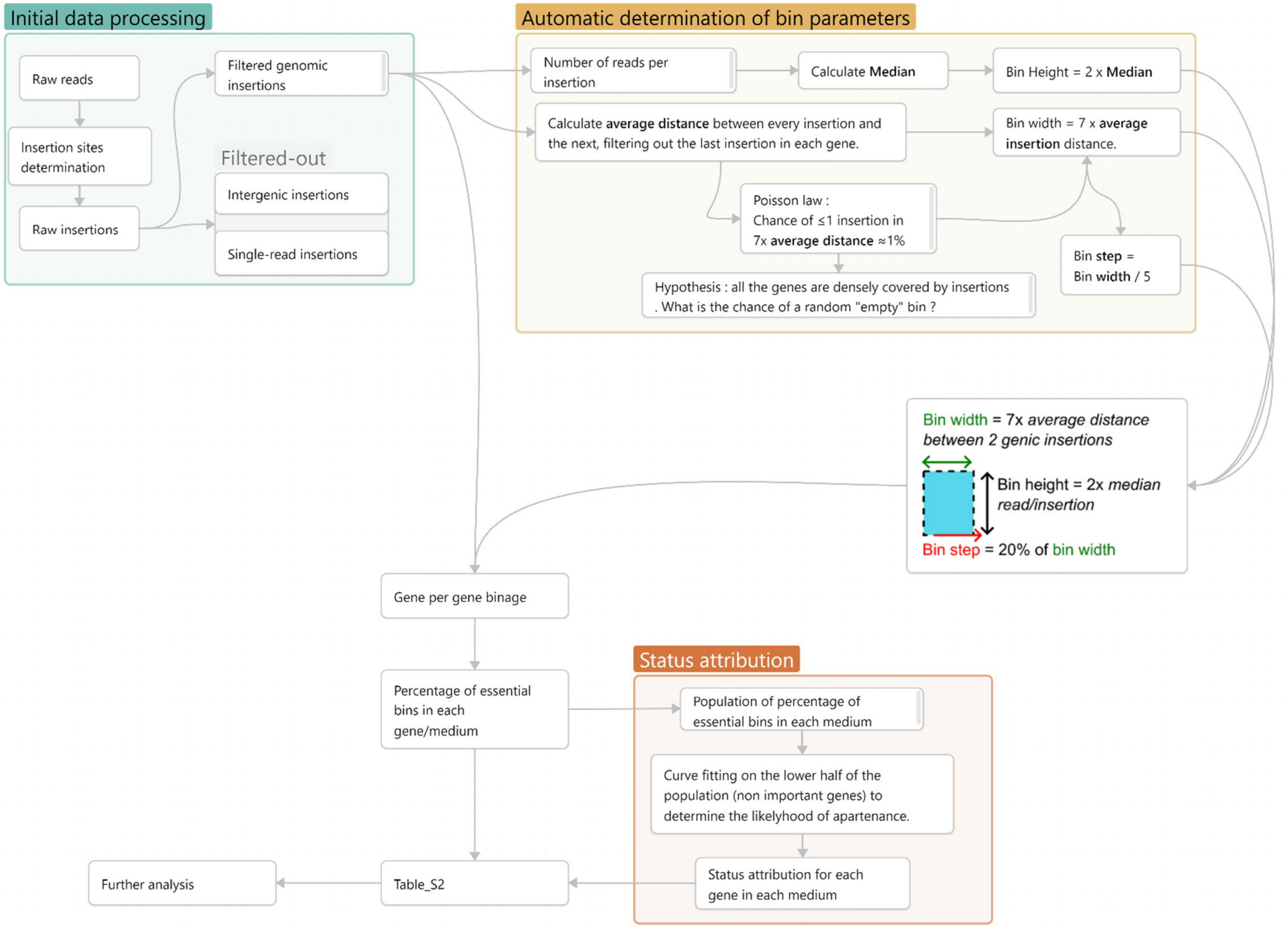

Supplementary Figure 2: Visual representation of the *fiScore* analysis procedure. To evaluate gene importance, the procedure constists of three major steps: initial data processing, automatic determination of bin parameter, and status attribution. Raw reads used as input are pooled fastq files of all replicates for a given passage and experimental condition.

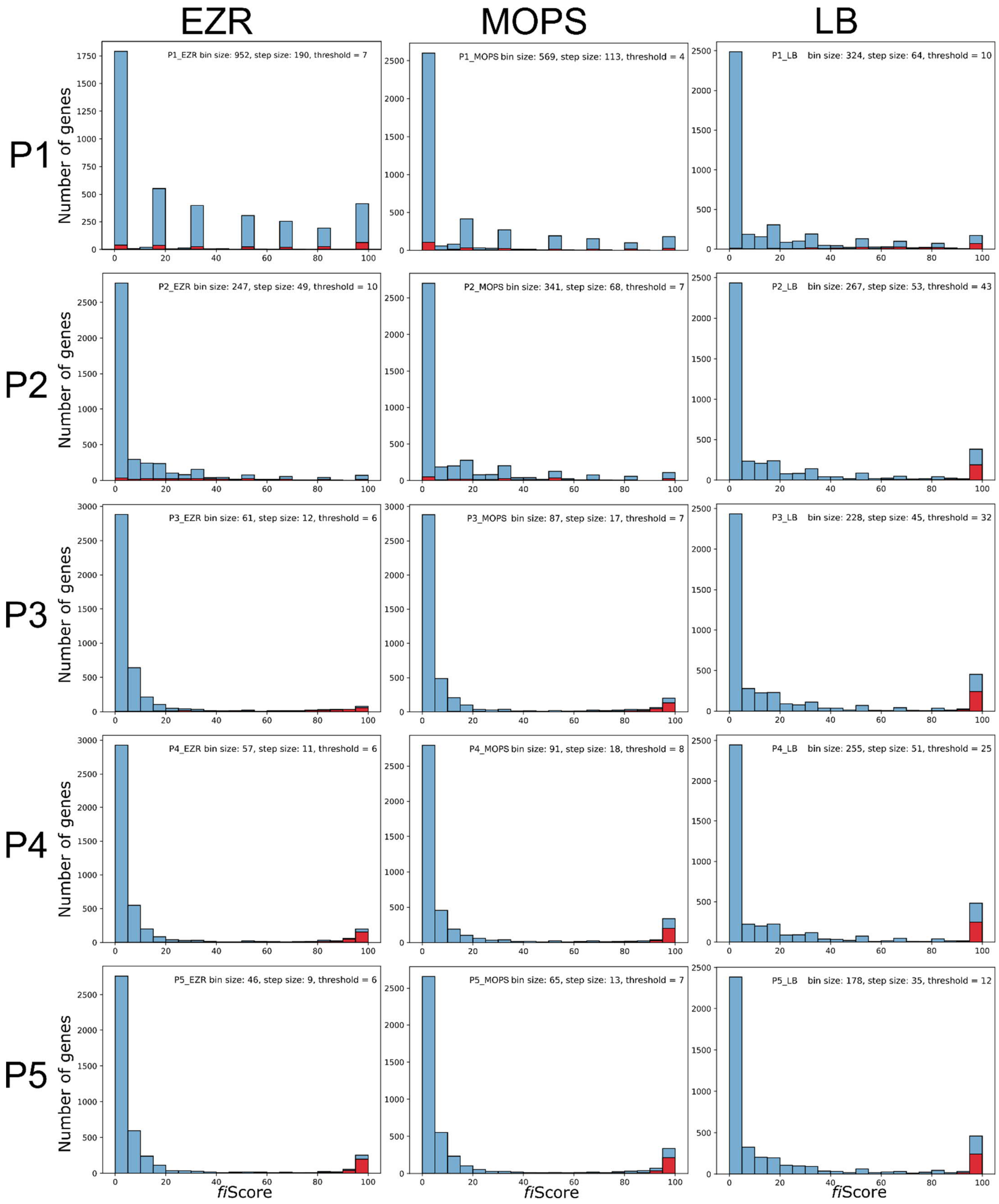

Supplementary Figure 3: Repartition of genes according to their *fiScore* across all passages and growth media. Automatically determined bin size and number of reads thresholds are displayed in each plot. A subset of high-confidence important genes (genes determined to be important in all three media and with a size > 1000 bp) are displayed as a red portion of bars for quality control.

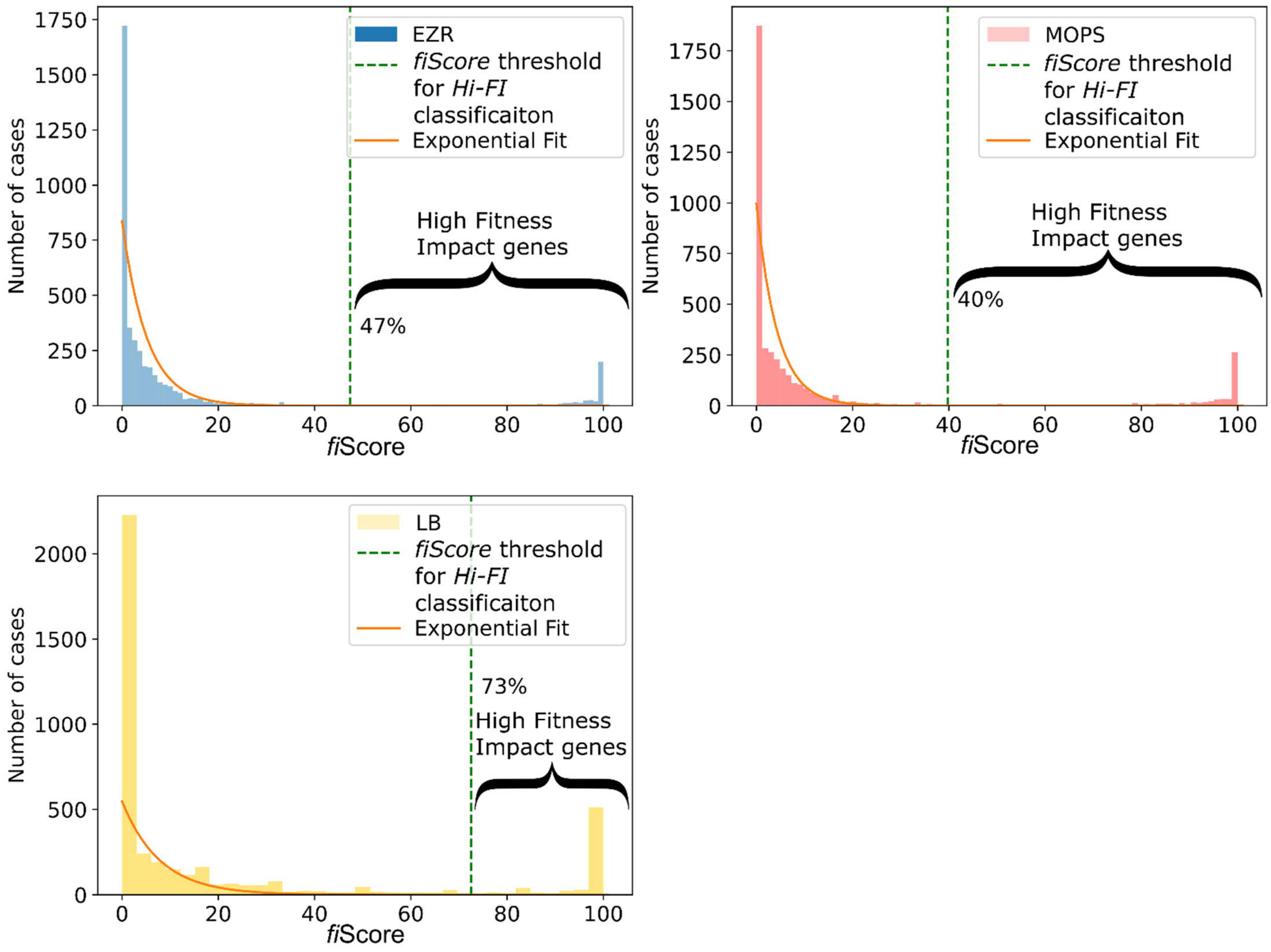

Supplementary Figure 4: Determination of High Fitness Impact genes using exponential fitting of the non impactinggene population. For each sample analyzed at passage 5 (from **Fig. S3**), the distribution of *fiScore*s per gene exhibited a bimodal pattern. Using the left-hand group of genes (*fiScore* < 80), which represents genes accumulating transposon insertions, we estimated their distribution with a fitted exponentially decreasing curve per medium. Utilizing this curve, we determined the percentage of bins required to have a probability < 0.01 % of association with the non-impacting population, indicated by the green vertical bar. Genes with *fiScores* above this threshold were labeled as High Fitness Impact genes. The relatively higher *fiScore* required for classification as High Fitness Impact in the LB condition is caused by a lower insertion density in this condition which in turn causes the bins to be wider than in the other experiments and more prone to insertion noise.

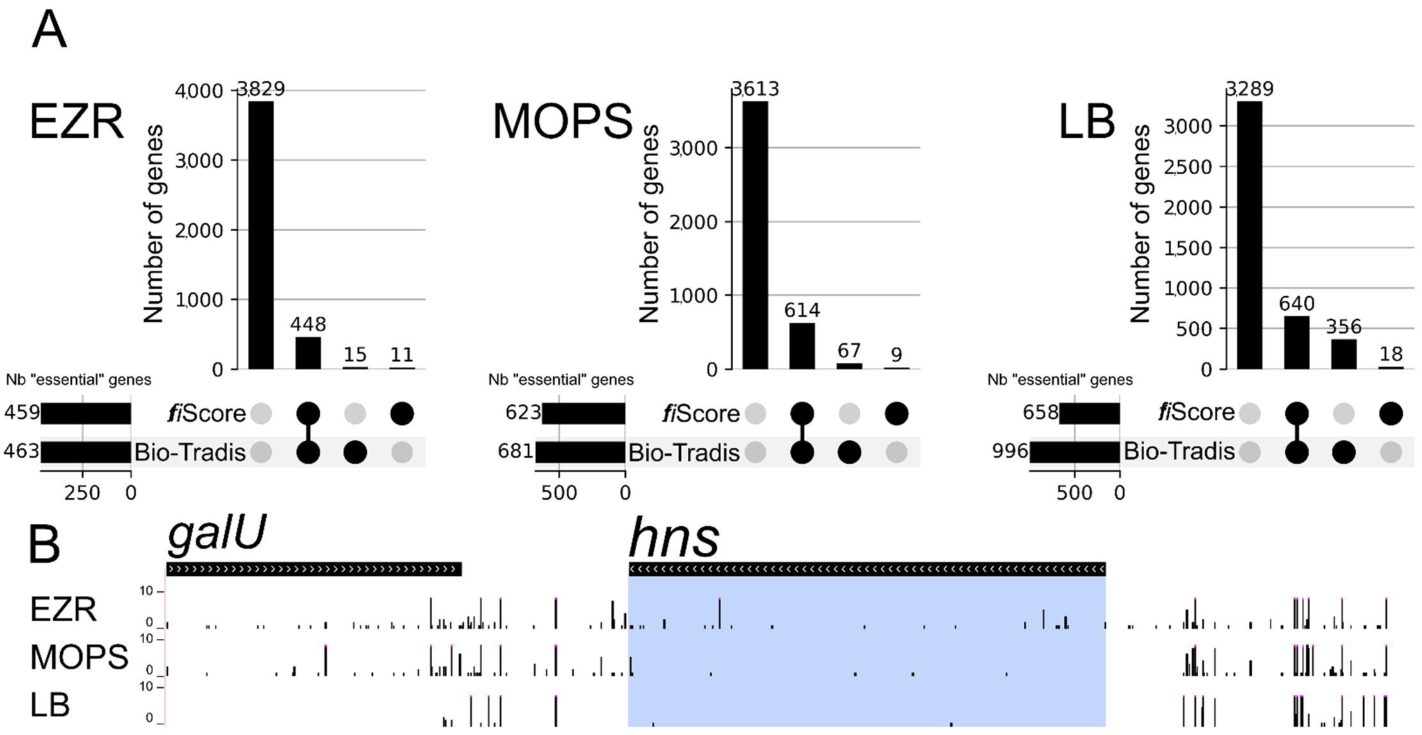

C	Common	Considered Essential or Ambiguous by BioTradis Only	Considered *Hi-FI* by *fiScore* Only
**EZR**	**4261 (99.39%)**	15<br>ybeD, sucB, rpmF, sdsN, dcd, ypeD, ibsC, yqgD, yrbN, trkA, ytiE	11<br>aceF, hemH, pflA, mukE, hns, sapA, ortT, ihfA, lapC, acpS, rnlB, higA, ilvX, efp, leuX,
**MOPS**	**4259 (98.23%)**	67<br>dnaJ, leuL, yaeP, ispA, ybaM, lipA, ybeD, rsfS, ybeY, ybgU, yljB, clpA, mukB, mepK, rmf, lpxL, rpmF, bhsA, mfd, ymfI, IS1414, rssB, pspA, hrpA, ortT, rnt, yoaL, zwf, dsrA, yeeA, asmA, cysP, ndk, iscU, acpS, rseA, rseD, ssrA, rnlB, micA, relA, rppH, ygfZ, yhaL, yrbN, rapZ, arcB, zapG, argR, tusB, tusC, secB, ysdE, ysdD, wecE, wecF, esrE, tatA, trkH, cpxR, metJ, aceK-int, hflX, ytgB, leuX, ryjB, yjjY	9<br>yahV, chiX, ychT, ruvA, yojO, yqcG, ilvL, cyaA, ytgA
**LB**	**4117 (91.31%)**	**356**<br>*thrA, nhaA, carA, carB, fixC, apaH, pdxA, mraZ, aceE, mrcB, yadS, glnD, gloB, rnhA, prpB, lacZ, lacI, frmR, yaiY, queA, pgpA, yajQ, yajR, cyoE, cyoB, hupB, ppiD, priC, ybaK, purE, ybdZ, fepG, fepB, ybdD, rnk, citX, lipB, dacA, ybeZ, fur, ybfE, seqA, pxpA, nei, sdhX, mngR, ybgC, cpoB, nadA, aroG, galM, modC, pgl, bioF, moaB, dinG, fiu, moeA, deoR, rimK, ybjN, artQ, ybjQ, amiD, clpA, dmsA, dmsB, pflA, pflB, elyC, mepK, pncB, ssuD, uup, pqiB, ycbZ, ompA, hyaC, hyaD, cbpM, rutC, rutB, rutA, putA, efeB, opgG, opgH, yceK, pyrC, flgM, flgJ, flgK, flgL, fabF, pabC, mltG, ycfH, ycfL, thiK, nagZ, mfd, potB, pepT, phoQ, minE, dadA, dadX, dhaL, dhaK, ychF, dauA, narH, narI, rssB, oppC, oppD, oppF, yciU, yciC, trpD, rluB, sohB, pyrF, ycjT, tyrR, fnr, ldhA, paaH, paaI, paaJ, aldA, trg, opgD, ydcO, ydcV, patD, ddpX, hipA, lsrK, lsrA, lsrD, tam, rspR, ynfE, ynfG, dmsD, bidA, mlc, pntA, rstB, tus, fumC, manA, rsxG, dtpA, gstA, slyA, ydhI, ydhJ, sodC, ydhL, lhr, purR, punR, punC, mdtK, ydhT, ydhX, sufS, sufA, ydiJ, ydiS, aroH, btuD, ydiZ, astB, astD, astA, ynjA, ynjB, nudG, topB, selD, ydjA, yeaC, yoaE, yoaL, manX,*	**18**<br>*eyeA, ffs, appX, ymdF, ymgK, ychT, fnrS, rydC, sokB, yobI, rseX, yoeJ, yfiS, sibC, yhgO, yriB, ytgB, thrL*

		mntP, mgrB, htpX, rsmF, yebW, ryeA, yebY, yebG, eda, zwf, pykA, lpxM, znuC, yebC, nudB, cmoA, cmoB, flhB, flhC, yedK, fliJ, fliN, yodD, dsrA, vsr, dcm, yedJ, tsuB, hisL, hisC, hisH, mdtA, mdtB, baeR, yegW, thiM, rcnR, mrp, mlrA, yehW, yohF, yeiW, bcr, rsuA*, ccmG, ccmD, ccmA, napD, ada, ftp, yfaA, yfaE, glpT, arnE, menE, menH, ackA,* pta, yfcE, cvpA, truA, pdxB, yfcJ, aroC, argW, fryA, cysZ, cysK, cysM, cysA, cysW, cysU, murQ, eutA, eutN, hda, purN, yfgM, hcaF, glyA, glnB, qseG, glmY, srmB, yfiM, kgtP, rluD, rnlB, nrdI, luxS, srlR, gutQ, hypC, yqcG, ygdG, ygdB, ppdA, ygdT, ygfX, gcvH, *ygfB, tktA, gshB, yggS, hybD, exbD, ttdB, nfeR, ebgR, exuR, yqjD, yqjE, yhbQ, nlpI, rbfA, yhbE, rapZ, arcB, nanE, zapG, argR, rsmB, tusB, rpe, damX, gntR, ftsX, ftsE,* rsmD, tusA, gor, baxL, mtlD, waaQ, rpmG, pyrE, spoT, ysdD, mnmE, mioC, rep, wecB, *rffG, rffH, rffC, rffM, cyaA, yigA, mobA, polA, glnA, pfkA, hslU, thiS, purD, yjaA, pmrR, aspA, epmB, frdD, hflX, hflK, rnr, ulaR, pepA, ytiE, yjjV, serB*	

Supplementary Figure 5**: Validation of the *fi***Score method and comparison with BioTradis. A) Comparison of essentiality status attribution (black circles) after passage 5 data processed using either the BioTradis[1] methodology and the newly developed analysis procedure (***fi***Score). B) Example of a gene (hns) having discordant status between the two analyses in EZR (BioTradis = non-essential; *fi*Score = important), where integration of the read/insertion counts likely leads to the differential status. C) List of all genes with a differential essentiality status between the two approaches.

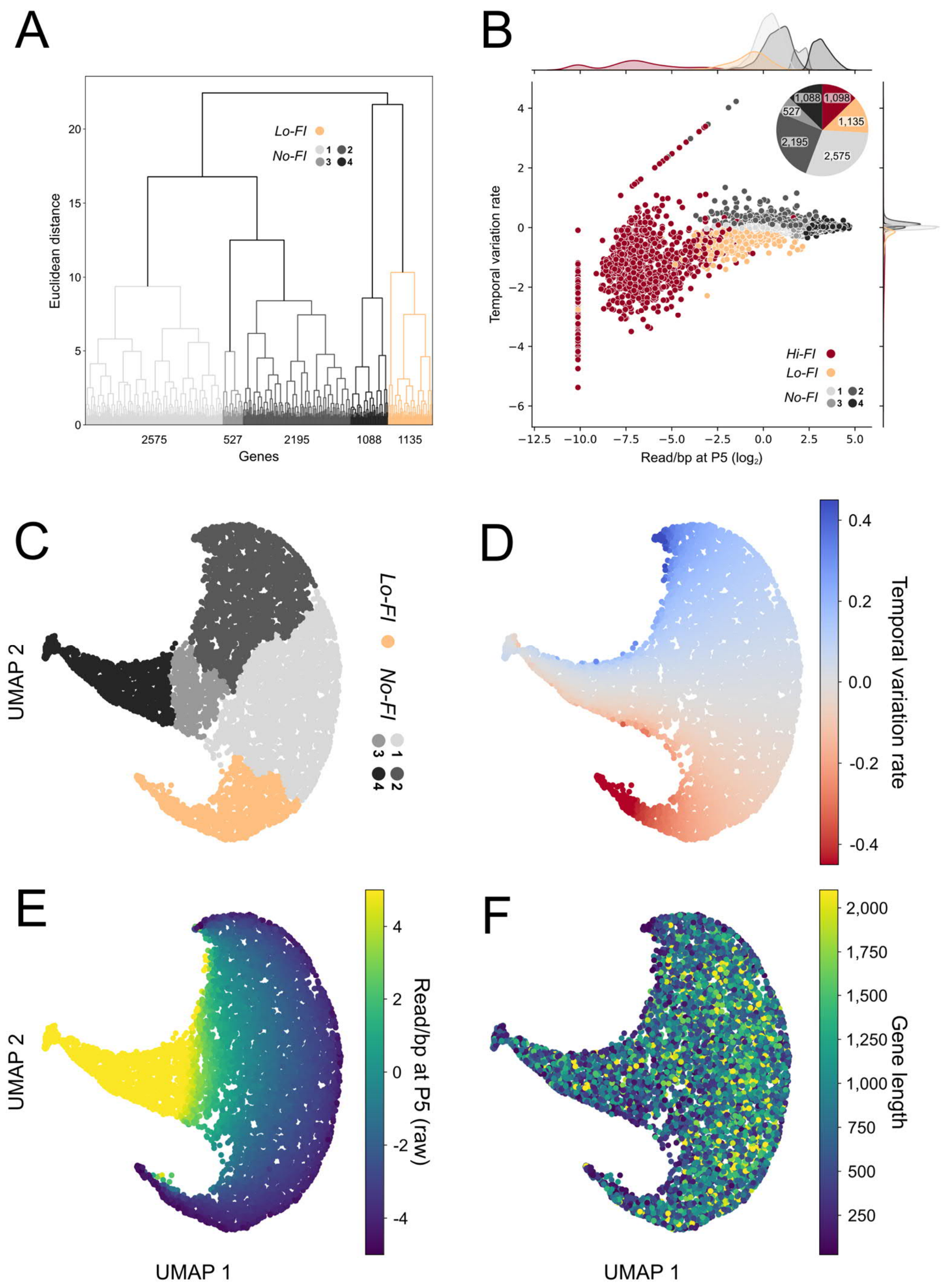

Supplementary Figure 6. Multidimensional classification of gene fitness impact across passages based on read abundance and temporal variations. A) Dendrogram depicting the hierarchical clustering supporting the choice of five clusters. Since the main driver of the clustering is the temporal variation rate, the neighboring of the *Lo-FI* and *No-FI* 4 clusters is not considered surprising. **B)** Temporal variation rate of all genes using normalized read counts across passages in either defined medium vs their read count per base at P5. Values for EZR and MOPS are plotted as visually indistinguishable individual dots (for a total of 8,618 genes). Genes are colored according to either their *Hi-FI* status (red) or their hierarchical cluster assignments (*Lo-FI* in orange or *No-FI* 1 to 4 in different shades of grey). Marginal density plots along the axes show the distribution of each metric across gene categories. The inset pie chart displays the absolute gene count per category, both medium combined. **C-F)** UMAP projections of non-*Hi-Fi* genes colored based on gene category (**C**), log_2 fold-change per passage (temporal variation rate) clipped at -0.45 to 0.45 **(D)**, scaled read count per base pair at P5 clipped at -5 to 5 **(E)**, gene length clipped at 95 % (suggesting gene length is not a primary driver of the observed clustering) **(F)**.

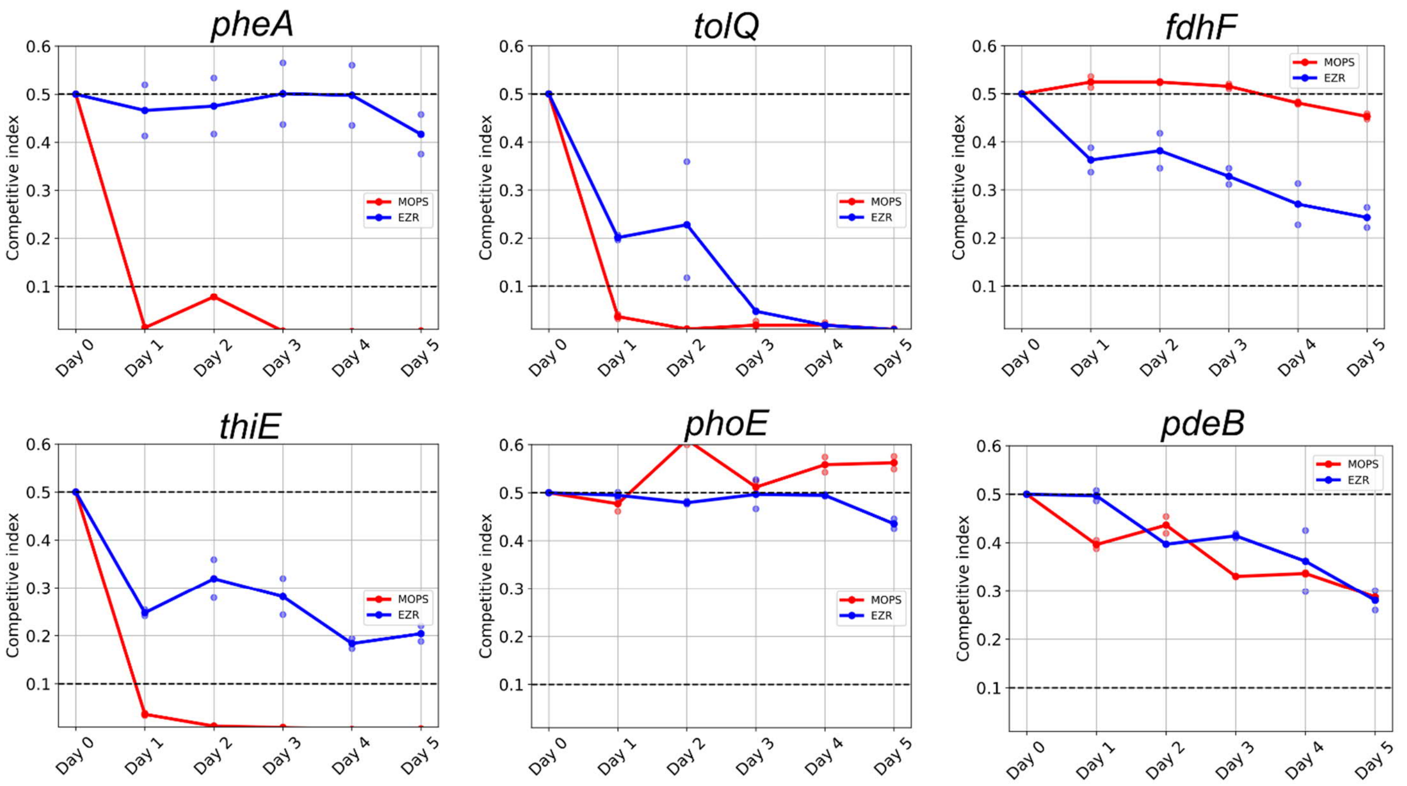

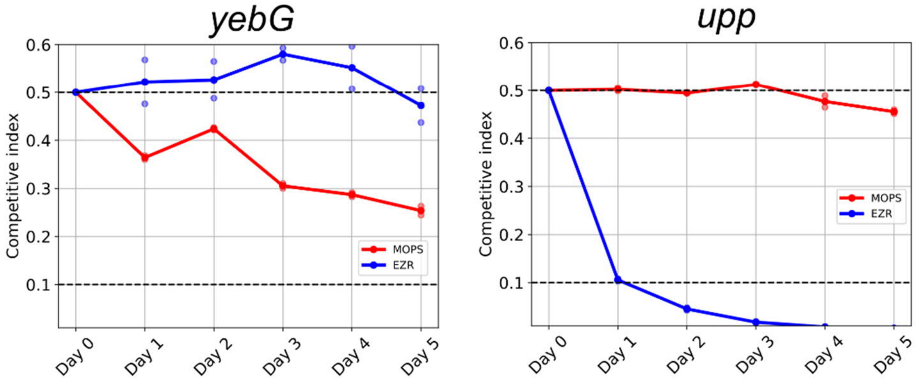

Gene	MOPS	EZR
pheA	*Hi-FI*	*No-FI*
tolQ	*Hi-FI*	*Hi-FI*
fdhF	*No-FI*	*No-FI*
thiE	*Hi-FI*	*No-FI*
phoE	*No-FI*	*No-FI*
pdeB	*No-FI*	*No-FI*
yebG	*Lo-FI*	*No-FI*
upp	*Lo-FI*	*Hi-FI*

Supplementary Figure 7: Competition assay between single-gene mutants from the Keio collection with different fitness impact status and the parental BW25113 wild type. After mixing equivalent amounts of cells using OD_{600} (Day 0), cell concentrations were measured every day in duplicates using a FACS machine (Days 1-5). Y-axis represents the competitive index calculated as follows: (number of mutants / total population). The table contains the fitness-based categories of each tested gene in each medium and the colors represent the concordance with the expected growth (green = accurate, orange = incorrect).

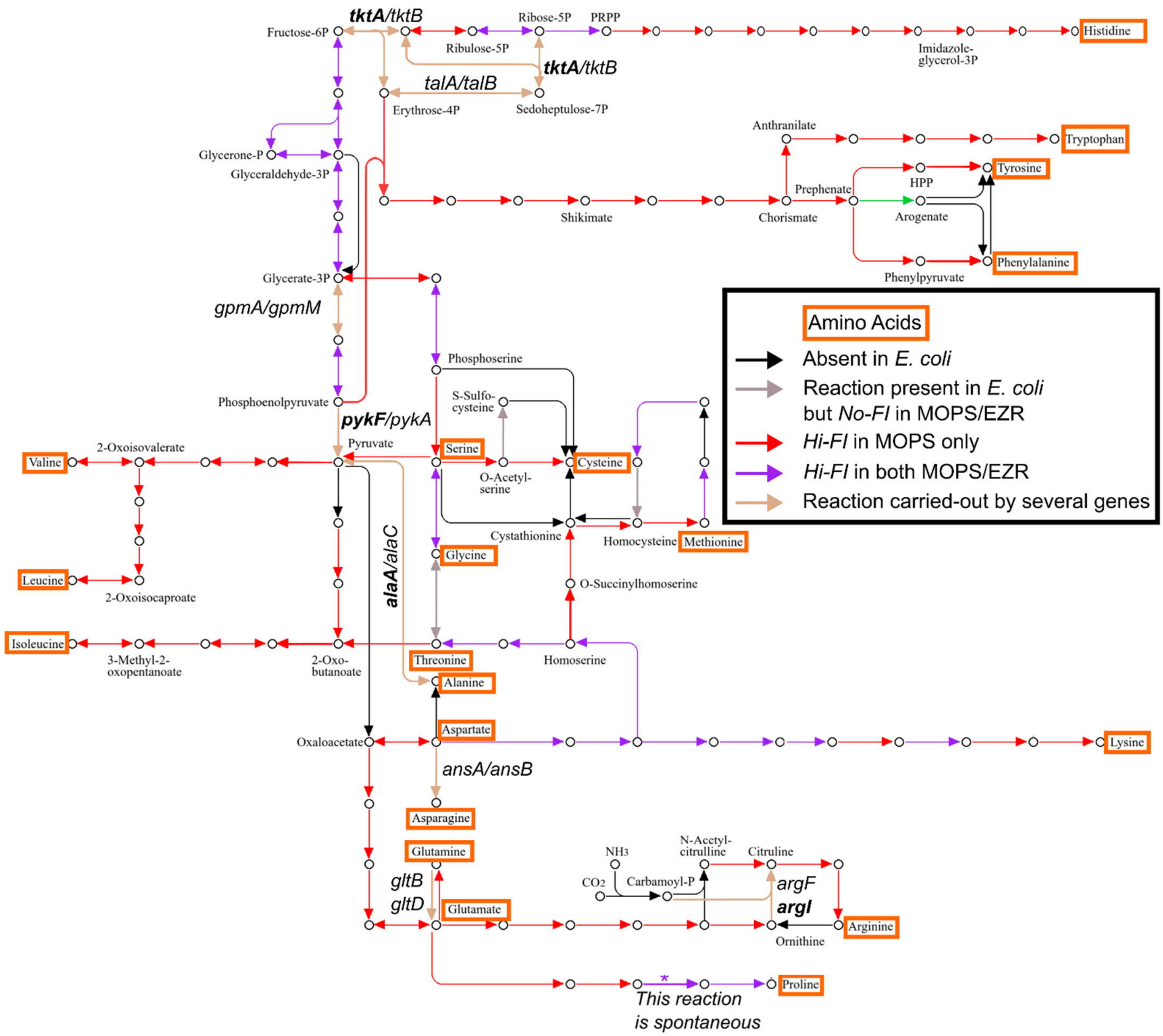

Supplementary Figure 8: Fitness Impact of the genes in the amino acid metabolism pathway in defined media. Based on the KEGG amino acid metabolism pathway map (eco01230), with manual recoloring. Reactions supported by multiple genes are annotated with the names of the known responsible genes. Among these, genes classified as *Lo-FI* in MOPS medium if any, are labeled in bold. Orange rectangles highlight amino acids within the pathway.

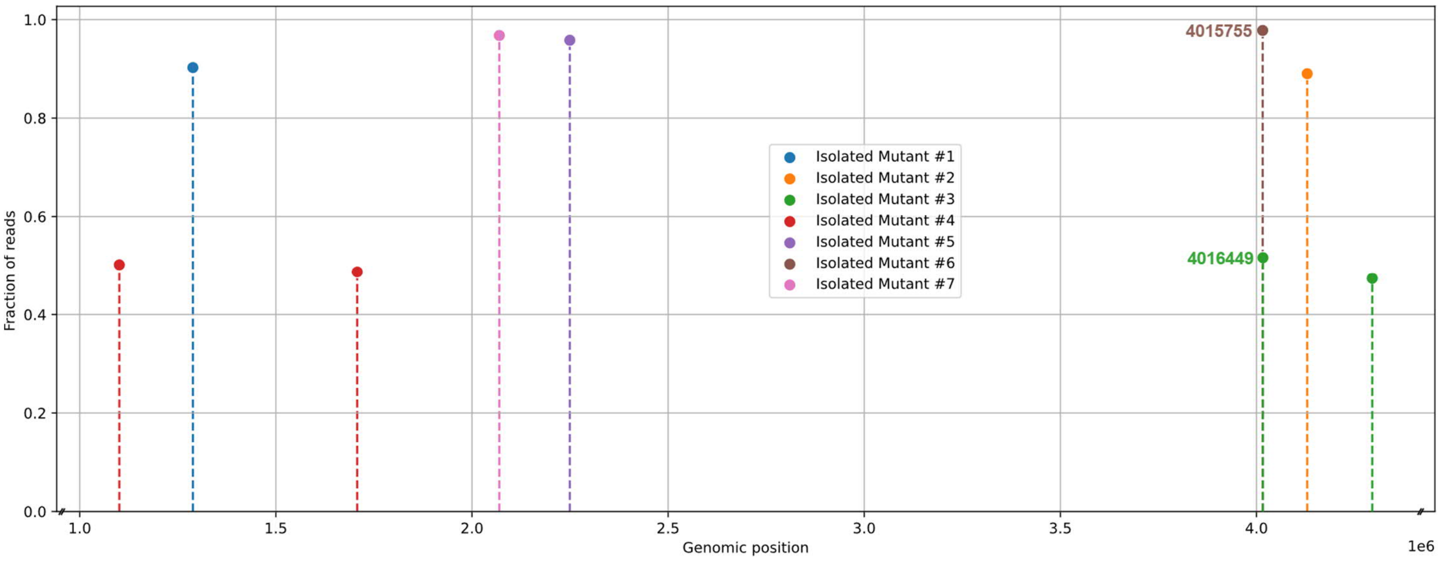

Supplementary Figure 9. Number and localization of the transposon insertions in isolated mutants. Lollipop chart displaying the fraction of reads associated with single base pair insertion positions in seven different isolated mutants. Artifactual sites backed by a signal under 5 % are not displayed.

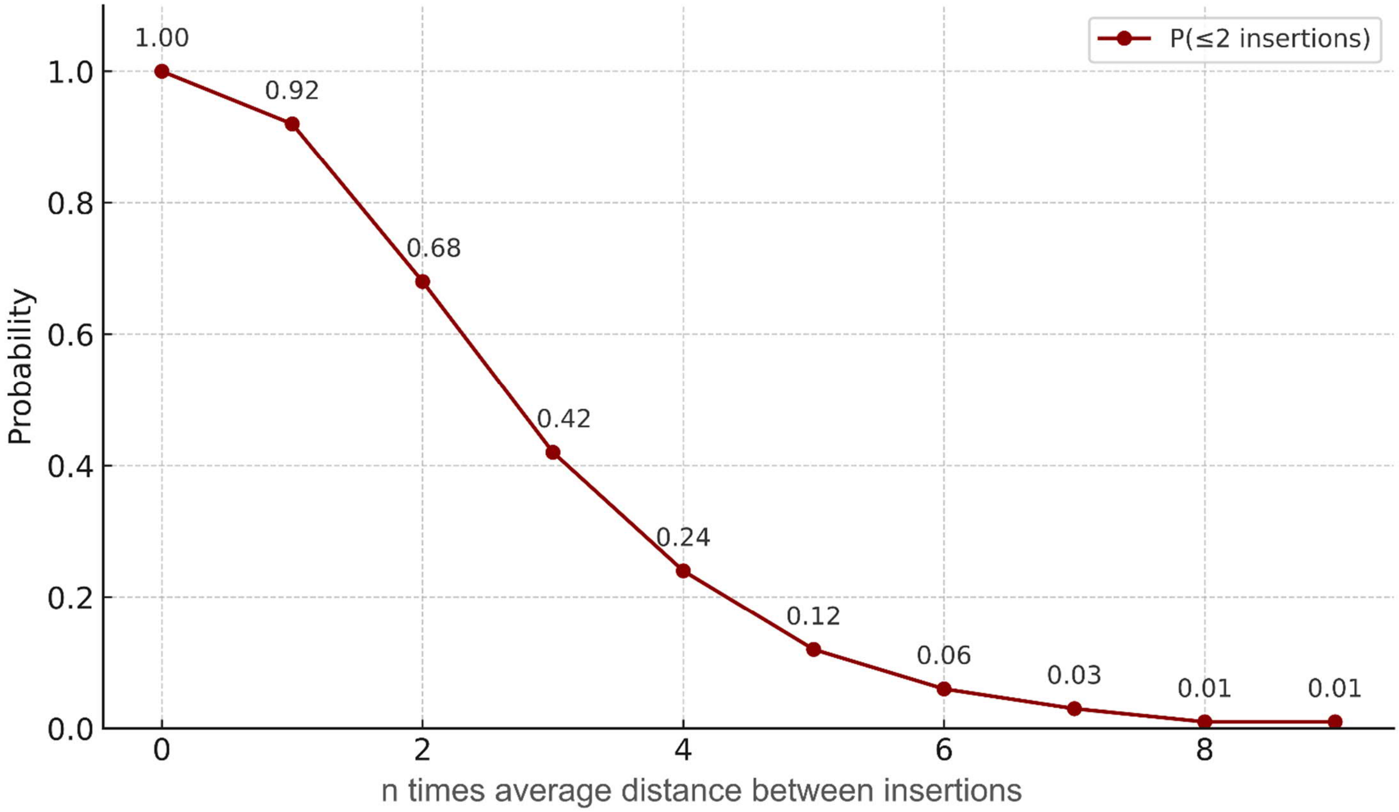

Supplementary Figure 10. Probability of observing ≤ 2 insertions in bins of varying length. The x-axis represents the bin size expressed as a multiple of the average insertion distance. The y-axis shows the probability of observing ≤ 2 insertions by chance in such a bin. Data point labels indicate the exact probability values.
